# Who Need Eye-Glasses

**Published:** 1881-06

**Authors:** 


### WHO NEED EYE GLASSES.

[From an Exchange.]

But there are only two kinds of eyes which need glasses. There are persons whose eyes are prismatic, that is, they see everything out of curve. Others are oversighted in one orb and nearsighted in the other. Others are deficient in the power of accommodation, that is, they do not readily "get used to it" when passing from a dark to a lighted room, or vice versa. Indeed the human eye is capable of as many metamorphoses as the soul in the old Greek philosophy, and the great wonder is that some people can see at all whose eyes are apparently as perfect as anyone's. For all these multiform weaknesses and irregularities, glass is the only remedy, and consequently its adaptation is a matter of scientific study. There are, besides thirty or forty convex and sixteen concave lenses (with varying focal distance), plano-convex, plano-concave, double convex, double concave, periscopic convex, periscopic concave and combinations of any or all of these to suit the peculiarities of a thousand individual eyes. Some notion can be got from this of the folly of intrusting the eyes to irresponsible hucksters, some of whom do not know a concave from a convex lens and hardly any of whom can give an inquirer the least optical information. It appears also to be folly for a person not versed in the physiology of the eye and the laws of optics to even attempt by himself to fit his eyes with glasses.

